# The combined effect of erythropoietin and granulocyte macrophage colony stimulating factor on liver regeneration after major hepatectomy in rats

**DOI:** 10.1186/1477-7819-8-57

**Published:** 2010-07-07

**Authors:** Ioannis Vassiliou, Evangelos Lolis, Constantinos Nastos, Aliki Tympa, Theodosios Theodosopoulos, Nikolaos Dafnios, George Fragulidis, Matrona Frangou, Agathi Kondi-Pafiti, Vassilios Smyrniotis

**Affiliations:** 1 Experimental Surgical Unit, 2nd Department of Surgery, Medical School, University of Athens, Aretaieion Hospital, Athens, 11528, Greece; 2 Department of Pathology, Medical School, University of Athens, Aretaieion Hospital, Athens 11528, Greece

## Abstract

**Background:**

The liver presents a remarkable capacity for regeneration after hepatectomy but the exact mechanisms and mediators involved are not yet fully clarified. Erythropoietin (EPO) and Granulocyte-Macrophage Colony Stimulating Factor (GM-CSF) have been shown to promote liver regeneration after major hepatectomy.

Aim of this experimental study is to compare the impact of exogenous administration of EPO, GM-CSF, as well as their combination on the promotion of liver regeneration after major hepatectomy.

**Methods:**

Wistar rats were submitted to 70% major hepatectomy. The animals were assigned to 4 experimental groups: a control group (n = 21) that received normal saline, an EPO group (n = 21), that received EPO 500 IU/kg, a GM-CSF group (n = 21) that received 20 mcg/kg of GM-CSF and a EPO+GMCSF group (n = 21) which received a combination of the above. Seven animals of each group were killed on the 1st, 3rd and 7th postoperative day and their remnant liver was removed to evaluate liver regeneration by immunochemistry for PCNA and Ki 67.

**Results:**

Our data suggest that EPO and GM-CSF increases liver regeneration following major hepatectomy when administered perioperatively. EPO has a more significant effect than GM-CSF (p < 0.01). When administering both, the effect of EPO seems to fade as EPO and GM-CSF treated rats have decreased regeneration compared to EPO administration alone (p < 0.01).

**Conclusion:**

EPO, GM-CSF and their combination enhance liver regeneration after hepatectomy in rats when administered perioperatively. However their combination has a weaker effect on liver regeneration compared to EPO alone. Further investigation is needed to assess the exact mechanisms that mediate this finding.

## Introduction

Liver has the unique capacity to regain its original and optimal mass after partial hepatectomy [1]. However the risk of immediate postoperative hepatic failure, especially if the procedure is performed in patients with a diseased liver, still represents a barrier to the extent of hepatectomy that can be attempted. The identification of factors that enhance liver regeneration and their clinical implication could reduce the morbidity and mortality associated with liver surgery.

However, liver regeneration is a complex phenomenon and the implicated mechanisms are not yet fully understood and clarified. It is well known that mature hepatocytes can replicate [1], representing the main mechanism of hepatocyte production during regeneration, as well as non-parenchymal cells that are located in the liver [2]. Bone marrow cells may also play a role in the generation of hepatocytes after liver injury, while it is known that many cytokines like IL-6 and TNFa and growth factors like TGFa, EGF and HGF are implicated in different stages of the regenerative process [3,4].

In studies that have been performed in the past, erythropoietin (EPO) has been shown to be produced by the regenerating liver after partial hepatectomy in rats [5] and erythropoietic foci have been found 24-72 hours after subtotal hepatectomy in rats [6]. EPO has been shown to have a positive effect on liver regeneration after hepatectomy in many studies [7,8]. In addition, EPO has been found to have a positive effect on liver regeneration after ischemia and reperfusion injury [9-11].

Granulocyte-Macrophage Colony Stimulating Factor (GM-CSF) is a cytokine that, besides the proliferation and differentiation of haemopoietic precursor cells, has additional effects on the functional properties of mature cells involved in inflammation and immunity [12]. It also enhances the functions of mature macrophages that are induced to secrete various cytokines including IL-6 and TNF-a, substances known to participate in liver regeneration [13]. GM-CSF has been used in the past, in order to stimulate liver regeneration following hepatectomy [14].

The combined administration of EPO and GM-CSF could possibly have a cumulative effect on liver regeneration. As this is an appealing intervention in order to enhance liver regeneration after hepatectomy, there are reports suggesting an antagonistic relationship between the two factors [15,16].

Aim of the present study is to compare the effect of the administration of EPO and GM-CSF alone or in combination on the acceleration of liver regeneration in rats after major hepatectomy.

## Materials and methods

Adult male Wistar rats weighing 200-250 gr each were obtained from the Hellenic Pasteur Institute (Athens, Greece) after the approval of the study protocol by Aretaieion Hospital Research Committee and the authority of the Athens prefecture for experimental protocols. They had free access to food and water and were kept in an air-conditioned room at 21°C with a 12-hr/12-hr light-dark cycle. The animals were fasted for 12 hr before the procedure and the same care continued in the postoperative period. Care and handling was in accordance with the National and European guidelines laboratory animal care.

Eighty four wistar rats were submitted to 70% major hepatectomy. The animals were assigned to 4 experimental groups: a control group (n = 21) that received normal saline, an EPO group (n = 21), that received EPO 500 IU/kg, a GM-CSF group (n = 21) that received 20 mcg/kg of GM-CSF and an EPO+GMCSF group (n = 21) which received a combination of the above. EPO, GM-CSF or normal saline were administered subcutaneously every day at 7 am for 8 days before the operation and for 2 days postoperatively.

For the induction of anesthesia 40 mg/kg ketamine (Ketalar 10 mg/ml) along with 1 mg/kg of atropine (atropine sulfate 1 mg/ml) were injected intramuscularly. Moreover, in a different side 5 mg/kg of Midazolam (Dormicum 15 mg/3 ml) diluted to 0.4 ml of normal saline 0,9% were also injected in order to maintain long lasting anesthesia of the animals undergoing liver resection. The surgery consisted of 70% partial hepatectomy according to the methods described by Higgins and Anderson [17]. The operations were performed between 9 am and noon. The resected liver was sampled for immunohistochemical study in order to evaluate if the factors that were administered for 8 days before hepatectomy had any effect on hepatocytes and to serve as self-control. Seven animals of each group were killed under anaesthesia by exsanguination on postoperative days 1, 3 and 7. Immediately after exsanguination the liver was removed for the study immunohistochemical study of regeneration.

Hepatic regeneration was evaluated by immunohistochemistry for Proliferating Cell Nuclear Antigen (PCNA) and Ki-67 [18]. Immunostaining of liver specimens was performed by using an anti-PCNA monoclonal antibody (PC-10, Dakopatts, Glostrup, Denmark). The three-step immunoperoxidase method using the Streptavidin-Biotin complex (Dakopatts) was performed, according to a procedure described previously [19]. Ki 67 was stained using a mouse anti-rat Ki-67 antibody (Dako, Denmark). Tissue sections were inspected at high power (x400 magnification) by two independent pathologists in a blind-coded manner. Positive nuclei were counted in 5-10 randomly chosen fields that approximate 1000 hepatocytes per section. The intensity of the staining was evaluated as negative, medium and high, the latter two being accepted as positive. Data were expressed as the percentage of cells that were positively stained.

The weight of the animals the day of surgery and the day of euthanasia was also recorded.

### Statistical Analysis

Data are expressed as mean ± SD. Differences between groups were analyzed by one-way analysis of variance (ANOVA), or if the data were not normally distributed by a Kruskal-Wallis ANOVA on ranks. Differences between time points of the same group were analyzed with univariate ANOVA. Bonferroni correction was used for post hoc multiple group comparisons. The level of statistical significance was defined as p < 0.05.

## Results

### Preoperatively

EPO pretreatment increased Ki 67 and PCNA expression preoperatively (p < 0.01). GM-CSF pretreatment as well as the combination of EPO and GM-CSF increased PCNA (p < 0.01), but not Ki 67 expression (p < 0.01).

### Postoperative day 1

On postoperative day 1 all rats had increased Ki 67 and PCNA expression (p < 0.05).

### Postoperative day 3

On postoperative day 3 all rats had increased Ki 67 (p < 0.05). PCNA was increased in the EPO and GM-CSF+ EPO groups, while there was no increase in the GM-CSF group.

### Postoperative day 7

One week after hepatectomy, hepatocytes showed increased expression of PCNA in all groups (p < 0.01), while Ki-67 was increased only in the EPO treatment group (p < 0.01).

**Figure 1:**
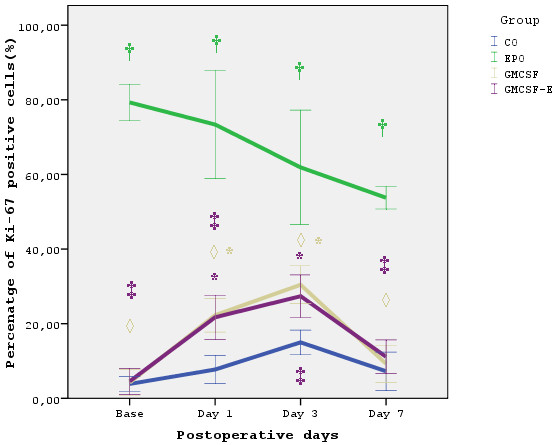
**Ki-67 expression**. Percentage of Ki-67 expression for each experimental group. Data are expressed as mean ± standard deviation. * p < 0.05 compared to baseline of the same timepoint. † p < 0.01 compared to baseline of the same timepoint. ‡ p < 0.01 compared to EPO group of the same timepoint. ◊ p < 0.01 compared to EPO group of the same timepoint.

**Figure 2:**
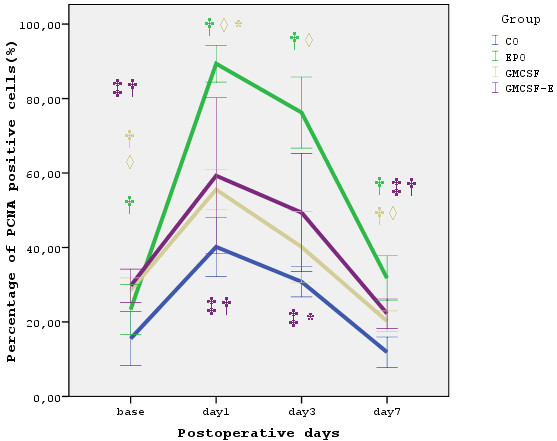
**PCNA expression**. Percentage of PCNA expression for each experimental group. Data are expressed as mean ± standard deviation. * p < 0.05 compared to baseline of the same timepoint. † p < 0.01 compared to baseline of the same timepoint. ‡ p < 0.01 compared to EPO group of the same timepoint. ◊ p < 0.01 compared to EPO group of the same timepoint.

In all postoperative days, the combination of EPO and GM-CSF failed to increase PCNA and Ki 67 staining to the extent that EPO alone did (p < 0.01). Both markers did not have any difference between the groups treated with GM-CSF and the combination of EPO and GM-CSF. In addition both Ki 67 and PCNA expression were significantly increased in EPO compared to GM-CSF treated animals in all post-operative days (p < 0.01). The results are summarized in Figures 1 and 2.

**Figure 3:**
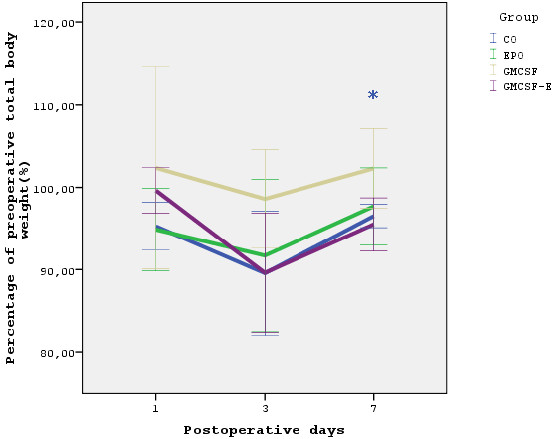
**Body weight**. Postoperative variation of total body weight. Data are expressed as mean ± standard deviation. * p < 0.05 compared to postoperative day 3 of the same group.

The percentage of postoperative total body weight variation did not differ significantly between groups as shown in Figure 3.

During the experiments 11 rats died, either due to hemorrhage or by immediate postoperative complications. These rats were excluded from the study and replaced by other animals.

## Discussion

Liver presents a remarkable capacity for proliferation after a partial hepatectomy and can precisely regulate its growth and mass to adjust its size. The exact mechanisms of stimulation and regulation of hepatic regeneration remain unclear. It is well known that various cytokines and growth factors and perhaps cell populations, other than hepatocytes are involved. Many different substances have been reported to stimulate liver cell growth in vivo and in vitro, including a number of known hormones, serum factors and some small nutrient molecules [1,2,4].

The discovery that EPO and its receptor play a significant biological role in tissues outside of the hematopoietic system has provoked significant experimental interest and fueled the exploration of additional actions of the hormone [20,21]. It is a member of the class I cytokines family and is considered a pleiotropic hormone. The EPO-specific receptor has been recognized in different cells, such as endothelial cells, epicardium, placenta, pancreatic islets, renal cells and defined areas of the brain [22]. Previous studies suggested that erythropoietin (EPO) was produced in rats by the regenerating liver [6] following partial hepatectomy and erythropoietic foci have been recognized 24-72 hours after subtotal hepatectomy in rats [6]. Kupffer cells seem to be the site of erythropoietin production after hepatectomy [5].

Angiogenesis seems to be a fundamental requirement for liver regeneration and its regulation. The modulation of endothelial cell proliferation or apoptosis has been shown to affect liver regeneration after partial hepatectomy in mice [23]. During liver regeneration the expression and activity of proapoptotic pathways is inhibited and after massive liver resection the activation of apoptosis is a major cause for failure of regeneration [24]. Recently EPO has been found to inhibit apoptosis after injury in various organs, like the brain [25], kidney [26] and the myocardium [27].

GM-CSF is a haematopoietic growth factor that apart from stimulating the proliferation and differentiation of myeloid bone marrow progenitor cells, also enhances the function of mature macrophages that are induced to secrete various cytokines including IL-6 and TNF-a [13]. It has been found to be a very potent immunostimulating agent by priming macrophages to produce cytokines, like TNF-a and IL-6 in blood of healthy humans as well as in blood of immunosupressed patients with sepsis and after cardiopulmonary bypass [28]. Within minutes after PH, Kupffer cells release cytokines, specifically TNF-a and IL-6 that are substantial for hepatocytes priming and preparation for replication [3]. Eroglu et al have already shown that GM-CSF promotes liver regeneration after hepatectomy in normal and cirrhotic livers [14].

The above mentioned experimental evidences prompted us to compare the effects of the administration of rhEPO, GM-CSF and their combination on liver regeneration following major hepatectomy. Although the effect of EPO in this setting has already been reported, there are few data on the effect of GM-CSF. In addition there are no data on the effect of their combination on liver regeneration. These two factors are thought to be mitogens and their combination should have a cumulative regenerating effect on the liver. However, Fatouros et al have reported that their combined administration seems to attenuate the beneficial role of EPO on intestinal anastomosis healing, which is similarly a mitotic process [16]. The major end-point of this study was to investigate if their combination has a synergistic or antagonistic effect on liver regeneration after major hepatectomy.

In our study we chose to evaluate the expression of two proliferation markers -PCNA and Ki 67-, as these have been shown to peak at different timepoints of the cell cycle, and their expression could vary depending on the stage of cellular duplication. They are sensitive markers of hepatocyte proliferation, which correlate well with the extent of regeneration [18]. In addition, they are have already been widely used for the study of liver regeneration and in particular for the study of the effects of EPO on liver regeneration.

Our study demonstrates that EPO administration had a positive effect on liver regeneration process after 70% hepatectomy by augmenting nuclear activity. This effect is noted even before any "triggering" for regeneration took place, as rats pre-treated with rhEPO showed increased expression of both Ki 67 and PCNA before hepatectomy was performed. This is in accordance with the literature, as Bockhorn et al have also demonstrated similar results. They reported that EPO preconditioning for three days can raise significantly the Ki-67 proliferation index and liver-to-body weight ratio of the normal liver [7]. In addition proliferation markers were increased after hepatectomy until 3 days on rats treated with rhEPO, similarly to our results [8]. Although the increase in our study is substantial, it is the result of a prolonged EPO pretreatment period (8 days). In addition, our results represent to total amount of hepatocytes stained, whether the staining was moderate or intense. As Ki 67 antigen is expressed during the whole cell cycle, it is uncertain whether the moderately stained cells are in the process of mitosis, or the antigen is still expressed in the cell after mitosis.

The dose of EPO administered in our study was 500 IU/kg and was administered subcutaneously. A wide variety of doses have been used by other authors [7-9,29,30]. We used the doses used by Fatouros et al in a study trying to compare the combined effect of EPO and GM-CSF on colonic anastomoses healing [15,16]. Generally they are considered low doses in this experimental setting. However we did not want to use higher doses as they have been shown to inhibit liver regeneration [29].

In our study, pre-operative GM-CSF administration resulted in increased hepatocyte proliferation before hepatectomy, as well as at postoperative days 1 and 7. Preoperatively only PCNA was over-expressed, and not Ki 67. This can be explained by the fact that these two markers of cellular proliferation do not correspond to the same cell cycle phase, as PCNA concentration seems to peak at the at the S phase of the cell cycle, while Ki 67 peaks later, during mitosis, in the M phase [18]. Eroglu et al showed increased hepatocyte proliferation 2 days after hepatectomy in rats were GM-CSF was administered. This effect however faded at the 7^th ^postoperative day. However in their study GM-CSF was administered immediately after hepatectomy, while in our study we pretreated animals for 8 days before hepatectomy and 2 days after [14]. The dose of GM-CSF administered was 20 mcg/kg as used by other authors [16].

On the other hand pretreatment with the combination of EPO and GM-CSF resulted in a weaker proliferative response compared with animals that were treated with EPO alone. Since EPO alone increased nuclear activity, it would seem logical that the combination group would have the same results. The fact that this group showed less nuclear activity than the EPO group, suggests perhaps a competitive action between the two growth factors. This is in accordance with the findings of other studies, where although EPO administration increased the tensile strength of colonic anastomoses postoperatively in rats, the combined administration of EPO and GM-CSF failed to show the same results [15,16]. Many possible mechanisms have been proposed in the literature. GM-CSF may play an antagonistic role on the EPO receptor as these hemopoietins have a high homology [31,32]. A competition between EPO and GM-CSF has been reported in cells of the marrow [33]. In addition, it has been shown that GM-CSF can modulate EPO effects in certain leukemic cell line models of hematopoiesis, modulating events at the transcriptional and signal transduction level, or decreasing mRNA levels of EPO receptor [34]. Finally concentrations of hemopoietins have been found to play a key role in the final effect on cellular response [35].

## Conclusions

In conclusion our data suggest that EPO and GM-CSF, when administered perioperatively in hepatectomy are able to accelerate liver regeneration. This can be added to the apparent beneficial effect of EPO in reducing blood transfusions that are associated with increased morbidity and might be of particular clinical interest in situations where hepatectomy is expected to result in significant liver failure and increased mortality. Future research can focus on the effect of these factors after hepatectomy when hepatic parenchymal disease coexists, as well as on the role of autologous transfusion inducing endogenous EPO production. Finally, the mechanisms involved in the inhibition of EPO by GM-CSF are the focus of our current research.

## Competing interests

The authors declare that they have no competing interests.

## Authors' contributions

VI, LE and SV conceived of the study, and participated in its design and coordination and helped to draft the manuscript. NC, TA and DN conducted the experiments. TT and FG participated in the design of the study and performed the statistical analysis, FM and KA performed the immunohistochemistry assays. All authors read and approved the final manuscript.
